# Calcium dysregulation combined with mitochondrial failure and electrophysiological maturity converge in Parkinson’s iPSC-dopamine neurons

**DOI:** 10.1016/j.isci.2023.107044

**Published:** 2023-06-07

**Authors:** Dayne A. Beccano-Kelly, Marta Cherubini, Yassine Mousba, Kaitlyn M.L. Cramb, Stefania Giussani, Maria Claudia Caiazza, Pavandeep Rai, Siv Vingill, Nora Bengoa-Vergniory, Bryan Ng, Gabriele Corda, Abhirup Banerjee, Jane Vowles, Sally Cowley, Richard Wade-Martins

**Affiliations:** 1 Oxford Parkinson’s Disease Centre, University of Oxford, Oxford, United Kingdom; 2 Department of Physiology, Anatomy and Genetics, University of Oxford, South Parks Road, Oxford OX3 7BN, UK; 3 Kavli Institute for Nanoscience Discovery, University of Oxford, Dorothy Crowfoot Hodgkin Building, South Parks Road, Oxford OX1 3QU, UK; 4 Radcliffe Department of Medicine, Division of Cardiovascular Medicine, University of Oxford, Oxford OX3 9DU, UK; 5 Department of Engineering Science, Institute of Biomedical Engineering, University of Oxford, Oxford OX3 7DQ, UK; 6 The James Martin Stem Cell Facility, Sir William Dunn School of Pathology, University of Oxford, South Parks Road, Oxford OX1 3RE, UK

**Keywords:** Pathophysiology, Cellular neuroscience, Stem cells research

## Abstract

Parkinson’s disease (PD) is characterized by a progressive deterioration of motor and cognitive functions. Although death of dopamine neurons is the hallmark pathology of PD, this is a late-stage disease process preceded by neuronal dysfunction. Here we describe early physiological perturbations in patient-derived induced pluripotent stem cell (iPSC)-dopamine neurons carrying the *GBA*-*N370S* mutation, a strong genetic risk factor for PD. *GBA-N370S* iPSC-dopamine neurons show an early and persistent calcium dysregulation notably at the mitochondria, followed by reduced mitochondrial membrane potential and oxygen consumption rate, indicating mitochondrial failure. With increased neuronal maturity, we observed decreased synaptic function in PD iPSC-dopamine neurons, consistent with the requirement for ATP and calcium to support the increase in electrophysiological activity over time. Our work demonstrates that calcium dyshomeostasis and mitochondrial failure impair the higher electrophysiological activity of mature neurons and may underlie the vulnerability of dopamine neurons in PD.

## Highlights

• *GBA-N370S* iPSC-dopamine neurons demonstrate a temporal sequence of PD related phenotypes
• Calcium signaling is disrupted in our PD model by reduced PLD1 and iPLA2
• Reduced mitochondrial efficiency resulted in reduced ATP production
• Our model *GBA-PD* system shows for the first time a reduced synaptic function

## Graphical abstract

**Figure:**
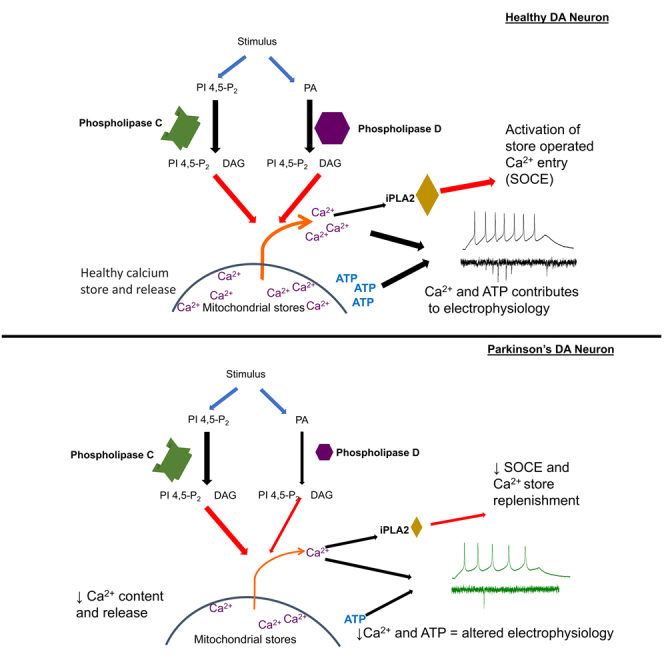

## Introduction

Mutations in the β-glucocerebrosidase (GCase) enzyme encoded by the *GBA* gene are the strongest common genetic risk factors for late onset Parkinson’s disease (PD). The GCase enzyme metabolizes glucocerebroside into glucose and ceramide within lysosomes.^1^ Cells carrying heterozygous *GBA* mutations such as N370S and L444P exhibit approximately 50% of wild-type GCase activity, increased GCase protein misfolding and endoplasmic reticulum (ER) stress which contribute to the risk of developing PD likely through a combination of loss and gain of function.^2^^,^^3^^,^^4^ Although the reduction in GCase activity impacts the autophagic/lysosomal pathway implicated in PD, evidence also supports the impact of GCase mutations on other neuronal functions. These include neurotransmitter release, neuronal ultrastructure, α-synuclein neuronal accumulation and release, and calcium handling, pointing toward a more complex role of *GBA* in PD pathology.^4^^,^^5^^,^^6^

Calcium is essential in a number of critical neuronal functions, such as neurotransmitter release, pacemaker activity and neuronal excitability,^7^^,^^8^^,^^9^^,^^10^^,^^11^ with calcium dysregulation well-described as a major cellular phenotype in PD.^12^^,^^13^ Calcium is also essential for ATP generation,^14^ important for neuronal functions such as ion channel regulation, neurotransmitter vesicle release/reuptake and synaptic receptor activation.^15^^,^^16^^,^^17^^,^^18^ Calcium and ATP are, therefore, at the center of the neuronal bioenergetic pathways which may underpin the preferential vulnerability of the dopamine neurons of the substantia nigra pars compacta (SNpc) in PD. SNpc neurons possess long unmyelinated arborized processes and display an order of magnitude more synapses (approximately between 1 and 2.5 x 10^6^ –fold more release sites) than other neuronal subtypes^19^ conveying a high metabolic demand.

Calcium dysregulation is evident in human iPSC-derived neuronal models of PD. The *GBA-L444P* mutation induces dysfunctional organelle calcium handling in neurons elevating both basal levels of somatic calcium and release of calcium from the ER, compared to controls.^6^ We have previously shown that *GBA-N370S* iPSC-derived dopaminergic neuronal cultures have significantly elevated levels of calcium-regulating proteins such as calreticulin.^4^ In addition, an aberrant ratio of the R-type voltage gated calcium channel Ca_v_2.3 (involved in dopamine neuron pacemaking) to the neuronal calcium sensor-1 (NCS-1; an important β subunit ion channel modulator^20^^,^^21^ and D2R receptor function^22^^,^^23^) results in alterations in excitability, pacemaking and internal calcium homeostasis, and drives dopamine neuron vulnerability and stress.^7^ Notably, no significant cell death attributed to *GBA* mutations was observed in any of these studies. It is likely, therefore, that calcium dysfunction alone does not cause the neuronal death seen in PD, but instead represents an early phenotype in disease pathogenesis which is important for neuronal vulnerability.

Here, we use iPSC-dopamine neurons carrying the *GBA-N370S* mutation to understand the early neuronal phenotypes in PD which precede cell death. Having defined early and late time points using electrophysiological assays in control iPSC neurons, we observed an organelle calcium release deficit localized at the mitochondria in young *GBA-N370S* iPSC-dopamine neurons. A reduction in ATP production was observed in these same neurons. Subsequently the increased bioenergetic demand, driven by the normal physiological increase in synaptic activity over time, combined with reduced mitochondrial efficacy associated with calcium dysfunction, resulted in reduced electrophysiological and synaptic activity in mature *GBA-N370S* neurons. We further identified aberrant expression of the critical calcium regulating proteins phospholipase D1 (PLD1), iPLA2 (*PARK14*) and synaptotagmin-1 which may underlie the observed phenotypes. In addition, reduced proximity of ER and mitochondria suggests a reduced calcium flux from the ER to mitochondria. Overall, our work demonstrates early Parkinson’s cell phenotypes which arise as calcium dysregulation, mitochondrial failure and synaptic activity converge in maturing neurons to drive neuronal vulnerability to disease. These pathways may represent suitable early therapeutic targets and inform on temporality of PD phenotypes important for identifying therapeutic windows before cell death and disease onset.

## Results

### Electrophysiological maturity of iPSC-dopamine neurons progresses over time

**Figure 1:**
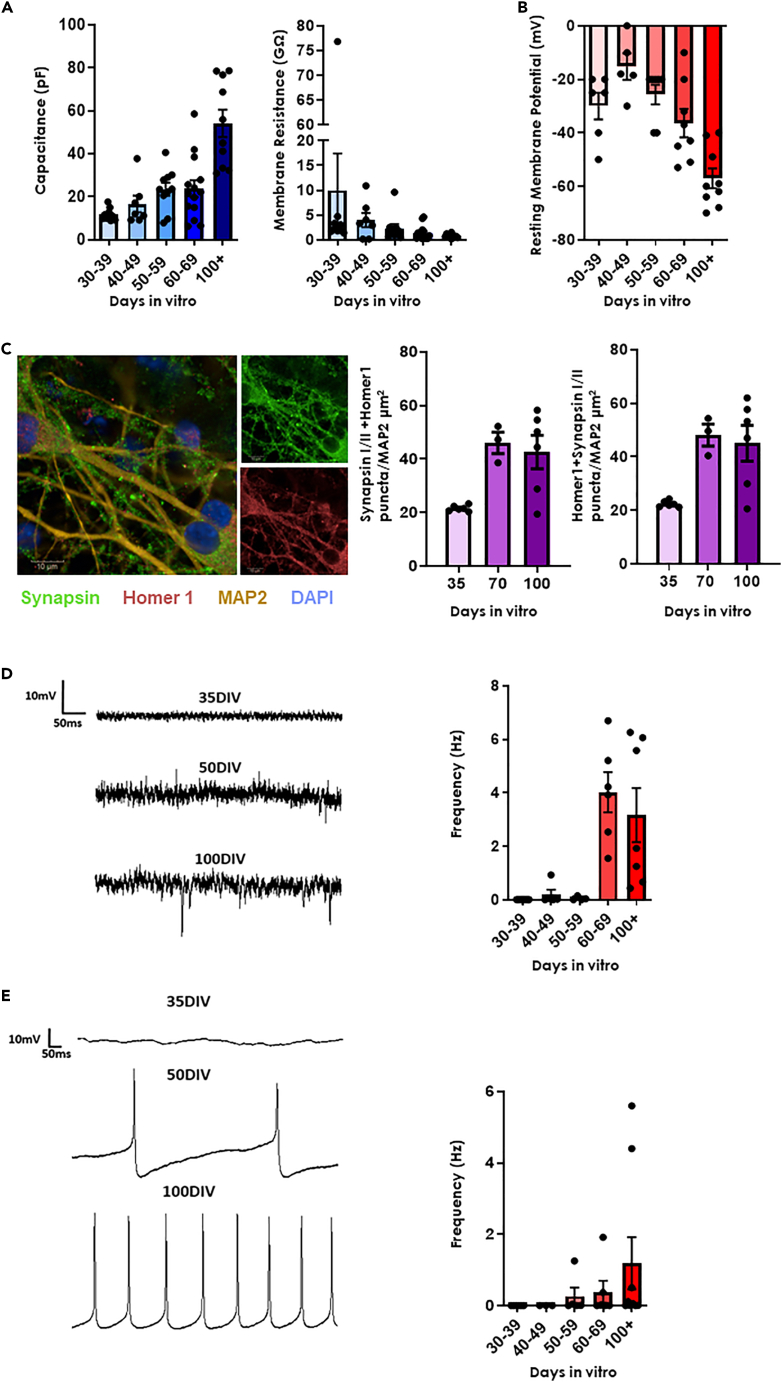
iPSC-dopamine neurons mature morphologically and electrophysiologically by 70 DIV (A) *Left*: Passive membrane properties measured using whole cell patch clamp from 2 different healthy control lines (Ctrl 1 & 5) over 3 differentiations illustrating increased capacitance over time separated into bins of 10 Days *In Vitro* (DIV). ***Right:*** shows decreased membrane resistance over same time period time. All data represented as mean ± SEM. (B) Resting membrane potential decreased over time and approached the normal potential (∼-40 mv-57 mV^32^^,^^33^) between 70 and 100 DIV. All data represented as mean ± SEM. (C) ***Left:*** Example immunocytochemical image from spinning disc confocal microscopy illustrating pre- (Synapsin) and post- (Homer1) synaptic markers, neuronal marker (Tuj1) and nuclear marker (DAPI) in a 70 DIV neuron (scale bar = 10μm). Middle: (Ctrl 4 & 5) quantification of Synapsin I/II apposition to Homer1 as a measure for synaptic number increased over time plateauing at 70DIV. ***Right:*** (Ctrl 4 & 5) quantification of Homer1 apposition to Synapsin I/II as a measure for synaptic number increased over time plateauing at 70DIV. All data represented as mean ± SEM. (D) ***Left:*** Example traces of spontaneous Excitatory Postsynaptic Currents (sEPSC) frequency at 35,50 and 100 DIV. ***Right:*** Average sEPSC frequency over time (Ctrl 1 & 5) reaching peak at 60–69 DIV. All data represented as mean ± SEM. (E) ***Left:*** Example traces of spontaneous pacemaker activity frequency at 35, 50 and 100 DIV. Right: Average pacemaker frequency over time (Ctrl 1 & 5). All data represented as mean ± SEM.

Previous work^24^^,^^25^^,^^26^^,^^27^^,^^28^ has shown that although iPSC-derived neurons display neuronal subtype-specific molecular markers at relatively early timepoints, synaptic function and neurotransmission take longer to evolve.^24^^,^^29^^,^^30^^,^^31^ To ascertain when iPSC-derived dopamine neurons display the electrophysiological activity critical to their physiological role, we performed whole cell patch clamp characterization on control iPSC-dopamine neurons generated using a previously described protocol^28^ to produce tyrosine hydroxylase (TH)-positive neurons (Figure S1). Electrophysiological measurements were obtained at several timepoints between 35 and 100+ days *in vitro* (DIV), and the data placed into 10-day blocks. In this manner, we ascertained the development of passive properties including an increase in cell capacitance (Figure 1A) and a concurrent decrease in membrane resistance (Figure 1A), over time. These likely represent an increase in neuronal arborization and insertion of functional ion channels into the membrane, respectively. Resting membrane potential (RMP) became more hyperpolarized over time (Figure 1B) until close to the resting membrane potential of a dopamine neuron *in vivo* (approximately between −40 and −57 mV^32^^,^^33^). Notably, an increase in staining of pre- and post-synaptic markers (Synapsin-1 and Homer, respectively) in apposition (Figure 1C), was observed, coincident with a dramatic increase in spontaneous excitatory postsynaptic currents (sEPSC; Figure 1D), confirming the formation of fully functional synapses at approximately 70 DIV. Furthermore, intrinsic pacemaking activity insensitive to inhibitors of network activity (CNQX, AP5 and bicuculine; Figure S2) was also seen at ∼70 DIV (Figure 1E). These features of fully functional dopaminergic neurons were accompanied by the presence of large A-type K_v_^34^^,^^35^ and hyperpolarization-induced membrane potential sag^36^^,^^37^^,^^38^ (Figure S2) demonstrating that these neurons could be considered electrophysiologically mature by 70 DIV. We therefore chose to use 35 DIV and 70+ DIV as timepoints to compare biological phenotypes at early and late stages of neuronal maturity, respectively.

### *GBA-N370S* iPSC-dopamine neurons display reduced mitochondrial calcium signaling

**Figure 2:**
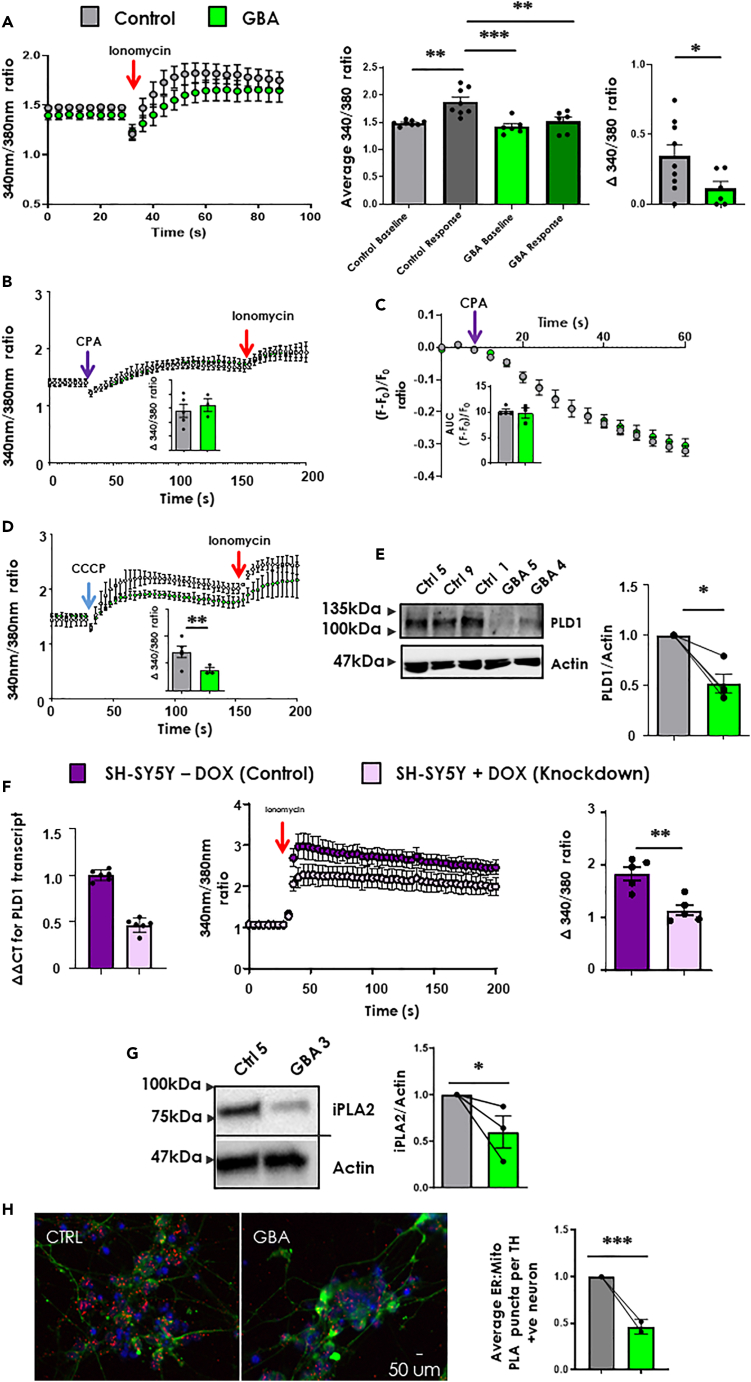
Decreased mitochondrial calcium release in *GBA-N370S* iPSC-dopamine neurons (A) *Left:* Average ionomycin evoked (red arrow) calcium release from 5 control (Ctrl 1,3,5,7&9) and 3 *GBA-N370S* (*GBA* 3,4,5) iPSC-dopamine neuron cultures over 4 differentiations, as measured by the ratiometric fluorescent dye Fura-2. ***Middle:*** average 30 s baseline and average peak response for control and *GBA-N370S* iPSC-dopamine neuron cultures (two-way ANOVA with Bonferroni’s post hoc testing α threshold 0.001, control (baseline versus response): p = 0.0011; control versus *GBA* (response versus baseline, respectively): p = 0.0004; control versus *GBA* (response versus response): p = 0.0051). ***Right:*** delta between baseline and average response representing magnitude of change in somatic calcium in response to ionomycin addition where negative values represent a negligible response and placed as zero (p = 0.0344 unpaired *t*-test with Welch’s correction). All data represented as mean ± SEM. (B) Control (Ctrl 1,4,7) and *GBA-N370S* (*GBA* 2,3,4) iPSC-dopamine neuron cultures over 2 differentiations were exposed to cyclopiazonic acid (CPA, 45 μM; purple arrow) causing a calcium leak from the Endoplasmic Reticulum (ER), with cell viability and calcium release confirmed by subsequent ionomycin addition (red arrow) 2 min post CPA application. *Inset:* average delta of calcium leak caused by CPA using three points at the maximum rate. Unpaired *t*-test with Welch’s correction and all data represented as mean ± SEM. (C) Calcium imaging using the ER-specific GCEPIAer sensor found no significant difference in ER calcium leak between *GBA-N370S* or control iPSC-dopamine neurons after treatment with CPA at 90 μM. (Ctrl 1,4,7 and *GBA-N370S* 2,3,6,8). *Inset:* area under the curve for each genotype. One-tailed Mann-Whitney test and all data represented as mean ± SEM. (D) iPSC-dopamine neuron cultures exposed to carbonyl cyanide *m*-chlorophenyl hydrazone (CCCP, 20 μM; blue arrow) released calcium from mitochondria, with cell viability and calcium release confirmed by subsequent ionomycin addition (red arrow) 2 min post CCCP application (Ctrl 1,4,7 and *GBA-N370S* 2,3,4). *Inset:* average calcium release because of CCCP using three points at peak calcium release (p = 0.002 unpaired *t*-test with Welch’s correction). All data represented as mean ± SEM. (E) ***Left:*** example western blot for Phospholipase D1 (PLD1; 120 kDa) ***Right:*** average PLD1 Optical density per differentiation in relation to control. Adjoining lines represent a differentiation (p = 0.0286 using Mann-Whitney U test). All data represented as mean ± SEM. (F) ***Left:*** qPCR data showing knocked down (KD) levels of PLD1 in a mixed SH-SY5Y population (points represent technical replicates with bars representing S.D.). ***Middle:*** Ionomycin induced calcium release in WT and PLD1 KD mixed population as measure by Fura-2. ***Right:*** Average delta of calcium release of baseline versus ionomycin response (p = 0.0026 by unpaired *t*-test). All data represented as mean ± SEM. (G) ***Left:*** example western blot for calcium-independent phospholipase A2 (iPLA2; 85 kDa) ***Right:*** average iPLA2 Optical density normalized to control. Each adjoining line represent a separate differentiation with points representing average control or *GBA-N370S* iPSC-dopamine neuron data for that differentiation (p = 0.0173 using Mann-Whitney U Test). All data represented as mean ± SEM. (H) *GBA-N370S* iPSC-dopamine neurons exhibit reduced ER/mitochondrial interaction by proximity ligation assay (PLA) puncta. Lines used: Ctrl 2,3,4,5&8 and *GBA-N370S* 1,3,4,5 over 2 differentiations. ***Left:*** example image for ER-Mitochondria-PLA. Red illustrates IP3R3 (ER) and VDAC1 (mitochondria) interaction; Green illustrates tyrosine hydroxylase and blue illustrates DAPI stain (scale bar = 50μm). ***Right:*** Average PLA puncta per TH positive neuron relative to control. Adjoining lines represent a differentiation (p = 0.000353 using random intercepts model). All data represented as mean ± SEM. All significances are indicated by asterisks: ∗p < 0.05, ∗∗p < 0.01, ∗∗∗p < 0.001, ∗∗∗∗p < 0.0001.

Acute intracellular calcium release from organelle stores such as mitochondria and endoplasmic reticulum (ER) is essential for neuronal functions such as synaptic plasticity and neurotransmitter release.^39^ We have previously shown that calreticulin, a protein important in calcium homeostasis in intracellular stores, was elevated in PD *GBA-N370S* iPSC-dopamine neurons.^4^ To investigate calcium signaling and release, control and *GBA-N370S* iPSC-dopamine neurons were assessed for calcium dysregulation using ratiometric calcium recordings. Using acute ionomycin stimulation known to result in organellar calcium release,^40^^,^^41^ we compared iPSC-dopamine neurons generated from healthy control iPSC lines, and from iPSC lines carrying the *GBA-N370S,* across multiple different differentiations. Mature neurons at 70+DIV of control but not *GBA-N370S* genotypes had a significant increase in somatic calcium concentration after ionomycin stimulation as detected using the ratiometric dye Fura-2. When the difference between baseline and maximal store release (at approx. 6–10 s post-stimulation) was compared we found that *GBA-N370S* iPSC-dopamine neurons released significantly less calcium than controls (∼50% reduction; p = 0.0344; Figure 2A). We then showed that this effect was present at earlier stages of neuronal development (35 DIV; Figure S3).

As ionomycin works on multiple intracellular calcium stores, including the ER and mitochondria but not lysosomes,^42^ we sought to identify which store was responsible for the release deficit to understand the impact of aberrant calcium homeostasis on neuronal dysfunction. We first demonstrated using our Fura-2 assay for cytoplasmic calcium that ER calcium leak did not differ between genotypes after inhibiting the SERCA pump with cyclopiazonic acid (CPA) (Figure 2B). To confirm this finding, we then investigated ER calcium leak using the genetically encoded ER calcium sensor GCEPIAer,^43^ a method which allows calcium dynamics to be measured specifically from the ER. Using the GCEPIAer sensor we found no significant differences in ER calcium leak between iPSC-dopamine neurons derived from individuals with the *GBA-N370S* mutation and those from controls after CPA treatment (Figure 2C). Finally, depolarization of the mitochondrial membrane potential using carbonyl cyanide *m*-chlorophenyl hydrazone (CCCP) showed significantly decreased calcium release from the mitochondria in iPSC-dopamine neurons derived from individuals with the *GBA-N370S* mutation compared to controls (Figure 2D).

### PLD1 and iPLA2 link aberrant mitochondrial calcium signaling and intracellular stores replenishment in *GBA-N370S* iPSC-dopamine neurons

Next, we identified potential mechanisms which may underlie the dysregulation of calcium homeostasis. We found that phospholipase D1 (PLD1; an important neuronal calcium signaling molecule^44^) was significantly decreased by 50% in *GBA-N370S* iPSC-dopamine neurons at the protein level (Figure 2E). To validate the involvement of PLD1 in calcium dysregulation we used CRISPR technology to knockdown PLD1 in the SH-SY5Y neuroblastoma cell line. Lentiviral transduction of gRNAs reduced PLD1 expression by 50% in SH-SY5Y cells (Figure 2E). Ionomycin stimulation of PLD1-knockdown (KD) cells resulted in a significant reduction in the amount of calcium released from intracellular stores into the cytoplasm as detected by Fura-2 compared to parental control cells (Figure 2F), phenocopying the calcium phenotype observed in *GBA-N370S* iPSC-dopamine neurons.

In addition to a reduction in the calcium release signaling protein PLD1, we observed a reduction in expression of calcium independent phospholipase 2 (iPLA2) protein in *GBA-N370S* iPSC-dopamine neurons (Figure 2G). The iPLA2 protein is important in replacing depleted organelle calcium stores via store operated calcium entry. Mutations in iPLA2 (*PARK14*) are associated with early-onset PD^45^^,^^46^ and lead to reduced replenishment of organelle calcium stores.^47^ The reduction in PLD1 and iPLA2 levels in *GBA-N370S* at 35 DIV iPSC-dopamine neurons suggests the neurons have deficits in both calcium release and calcium replenishment to stores.

In addition to disrupted signaling and replenishment of stores, the mitochondrial calcium deficit could result from decreased calcium flux from ER stores. The ER is the primary source of mitochondrial calcium so decreased ER-mitochondrial proximity would result in a reduction in mitochondrial calcium. Using the Proximity Ligation Assay with markers for mitochondria (VDAC1) and ER (IP3R3) we showed that there was a significant decrease in ER-mitochondrial proximity in neurons derived from *GBA-N370S* lines compared to controls (Figure 2H).

### *GBA-N370S* iPSC-dopamine neurons exhibit perturbed mitochondrial function and mitochondrial stress

**Figure 3:**
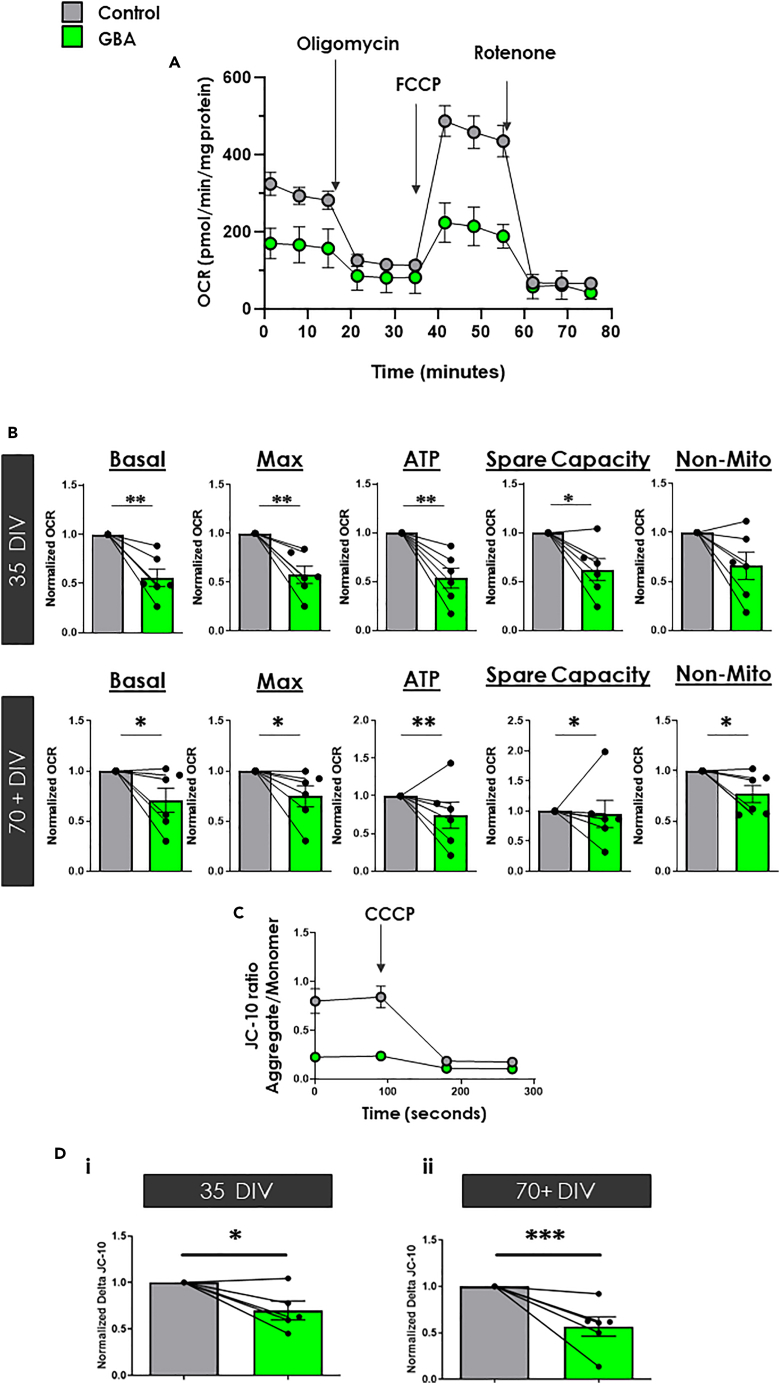
Reduced mitochondrial oxygen consumption in *GBA-N370S* iPSC-dopamine neurons persists over time (A) Example Seahorse trace from a single control (Ctrl 8) and single *GBA-N370S* line (*GBA* 1) illustrating oxygen consumption after various pharmacological modulation. All data represented as mean ± SEM. (B) Oxygen consumption rate attributed to basal respiration, maximal respiration, ATP production, Spare capacity and non – mitochondrial capacity (left to right) normalized to control iPSC-dopamine neurons per differentiation (Ctrl 1–8 and *GBA* 1–5) at 35 DIV (***upper***) and 70+ DIV (***lower***). All data represented as mean ± SEM. (C) Example JC-10 trace from a single control (Ctrl 5) and single *GBA-N370S* line (*GBA* 2) illustrating mitochondrial membrane potential as a function of lipophilic JC-10 dye localization after CCCP application. All data represented as mean ± SEM. (D) Delta fluorescent ratio from the JC10 assay measuring mitochondrial membrane potential. Each pair represents the average ratio normalized to average control for each differentiation (Lines used: Ctrl 1–6 and *GBA* 1–5). All data represented as mean ± SEM. All significances are indicated by asterisks: ∗p < 0.05, ∗∗p < 0.01, ∗∗∗p < 0.001, ∗∗∗∗p < 0.0001 using a random intercepts model.

Decreased calcium release from mitochondrial stores may reflect decreased mitochondrial calcium content, leading to decreased mitochondrial dehydrogenase enzymatic activity and mitochondrial dysfunction.^14^^,^^48^ To test whether *GBA-N370S* iPSC-dopamine neurons have dysfunctional mitochondria, we measured oxygen consumption rate (OCR) of basal, maximal, spare and ATP-associated respiration in control and *GBA-N370S* iPSC-dopamine neurons at the early (35 DIV) and mature (70+ DIV) time points using the Seahorse flux analyzer which measures the oxygen content in the media surrounding cells to assay oxygen consumption because of mitochondrial respiration. We observed decreased OCR of basal and maximal mitochondrial function in the *GBA-N370S* iPSC-dopamine neurons compared to controls at 35 DIV (Figures 3A and 3B). In addition, the spare capacity of *GBA-N370S* iPSC-dopamine neurons was significantly reduced (Figure 3B). As predicted, the amount of OCR attributed to ATP production was also significantly lower, meaning less ATP is generated by *GBA-N370S* iPSC-dopamine neurons per oxygen molecule (Figure 3B). This effect persisted over time and the OCR attributed to ATP generation in *GBA-N370S* iPSC-dopamine neurons remained significantly lower at the later timepoint (70+ DIV; Figure 3B) with basal, maximal and spare capacity OCR also significantly reduced. OCR values (basal, maximal, ATP and spare capacity) were markedly increased in both genotypes over time (i.e. between 35 DIV to 70+ DIV; data not shown), another indication of increasing maturity over time.

The reduced generation of ATP by mitochondria in *GBA-N370S* iPSC-dopamine neurons may reflect an inability to meet the bioenergetic demand as a result of decreased mitochondrial calcium, as opposed to a decreased ATP requirement of the neuron. Mitochondrial membrane potential (Δψm) drives ATP generation, with chronic changes to Δψm also indicating mitochondrial stress. To investigate this possible mechanism for dysfunctional mitochondrial OCR we measured Δψm using JC-10, a ratiometric dye which enters mitochondria and is used calculate mitochondrial membrane potential (Figure 3C). We observed decreased mitochondrial membrane potential in *GBA-N370S* iPSC-dopamine neurons at 35 DIV (p = 0.00221; Figure 3Di) and at 70+ DIV (p = 0.00759; Figure 3Dii), confirming the compromised mitochondrial function in PD neurons.

### Spontaneous quantal neurotransmitter release is compromised and synaptotagmin-1 levels are reduced in *GBA-N370S* iPSC-dopamine neurons

Taken together, these data support a hypothesis of unbalanced energy supply and demand as *GBA-N370S* iPSC-dopamine neurons mature. The increasing bioenergetic demand imposed by a rising, high frequency and persistent electrophysiological activity is likely unsustainable in the face of low levels of mitochondrial calcium and ATP production and will either result in a reduction of neuronal activity, or cell death.

**Figure 4:**
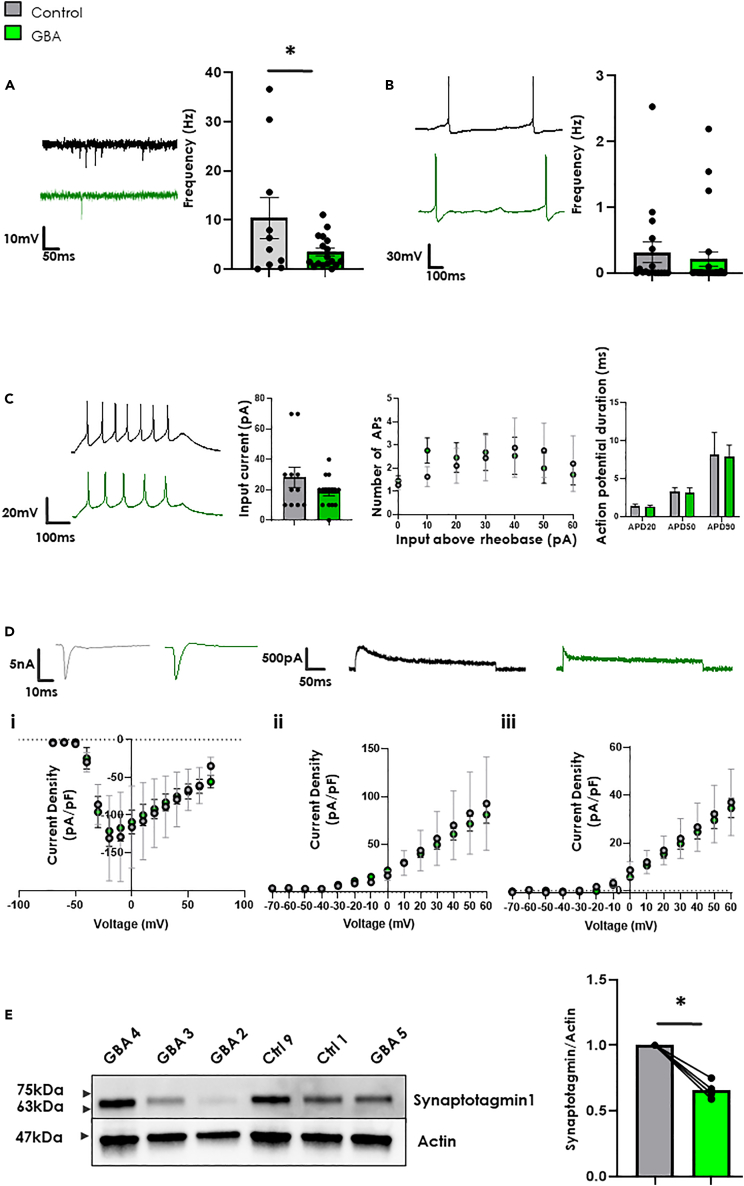
Reduced spontaneous Excitatory PostSynaptic Current (sEPSC) is not because of altered neuronal excitability or ion channel expression but decreased synaptic vesicle protein level (A) *Left:* Example traces of sEPSC from Control (***top***) and *GBA-N370S* (***bottom***) iPSC-dopamine neurons 70+ DIV. **Right**: mean activity showing significantly reduced sEPSC in *GBA-N370S* iPSC-dopamine neurons compared to controls (Two-tailed, Student’s *t* test; n = Ctrl 10, *GBA* 17; Ctrl 1,2,4,5,6 & *GBA* 1,3,4,5 across 4 differentiations). All data represented as mean ± SEM. (B) ***Left:*** Example traces of spontaneous pacemaking from Control (top) and *GBA-N370S* (bottom) iPSC-dopamine neurons 70+ DIV. ***Right:*** mean activity showing no significant change in in *GBA-N370S* iPSC-dopamine neurons compared to controls (Two-tailed, Student’s *t* test; n = Ctrl 19, *GBA* 26; Ctrl 1,2,4,5,6 & *GBA* 1,3,4,5 across 4 differentiations). All data represented as mean ± SEM. (C) ***Left:*** Example traces of induced action potentials from Control (***top***) and *GBA-N370S* (***bottom***) iPSC-dopamine neurons 70+ DIV. ***Middle Left:*** average minimum input required for single action potential (rheobase; n = Ctrl 11 GBA 13). ***Middle right:*** Input out graph of number of action potentials with increasing current input above rheobase. ***Right:*** Calculated 20, 50 and 90% action potential duration, a single AP at rheobase (two-way ANOVA; n = Ctrl 11, *GBA* 15). All data represented as mean ± SEM. (D) ***Left:*** IV curve of proposed sodium channel current density, ***Middle:*** IV curve of proposed A-type potassium channel current density, ***Right:*** IV curve of proposed delayed rectifier potassium channel current density. No significant difference was observed between any of these groups (Two-tailed, Student’s *t* test). All data represented as mean ± SEM. (E) ***Left:*** example western blot for Synaptotagmin (65 kDa) ***Right:*** average Synaptotagmin 1 Optical density normalized to control. Each adjoining line represent a separate differentiation with points representing average control (Ctrl 2,3,4 & 9) or *GBA-N370S* (*GBA* 1,3,4 &5) iPSC-dopamine neuron data for that differentiation (p = 0.0286 using Mann-Whitney U Test). All data represented as mean ± SEM. All significances are indicated by asterisks: ∗p < 0.05, ∗∗p < 0.01, ∗∗∗p < 0.001, ∗∗∗∗p < 0.0001.

To investigate this further, we compared electrophysiological recordings of *GBA-N370S* and healthy control iPSC-dopamine neurons. At 70+ DIV, when heightened spontaneous activity was observed in healthy neurons (Figure 1), we showed that the frequency of spontaneous excitatory postsynaptic currents (sEPSC) which are dependent on calcium and ATP^16^^,^^49^ were significantly reduced in *GBA-N370S* iPSC-dopamine neurons (Figure 4A). In contrast, the pacemaking activity characteristic of SNpc neurons was unchanged in *GBA-N370S* iPSC-dopamine neurons (Figure 4B). Induced action potentials (APs) showed no significant difference in frequency (with increasing input above rheobase) or duration, implying that the *GBA-N370S* iPSC-dopamine neurons are capable of heightened activity if forced (Figure 4C). Current density from voltage gated channels showed no significant difference between *GBA-N370S* and control iPSC-dopamine neurons (Figure 4D), also implying that *GBA-N370S* iPSC-dopamine neurons are capable of generating the same number of APs and action potential duration as control neurons if stimulated.

Calcium is responsible for regulation of neurotransmitter release and electrophysiological activity in neurons,^50^ a process controlled in part by specialized calcium binding proteins such as synaptotagmin-1. Synaptotagmin-1 tethers to and relocalizes synaptic vesicles based on their calcium bound state^51^ and is implicated in fast dopamine transmission. We found the levels of synaptotagmin-1 protein to be significantly reduced in whole cell lysates of *GBA-N370S* at 70+ DIV in iPSC-derived dopamine neurons which may explain the reduced neurotransmitter release and sEPSC frequency compared to controls (Figure 4E).

Taken together, these data show the reduction of the key calcium regulatory proteins PLD1, iPLA2 and synaptotagmin-1 in *GBA-N370S* iPSC-dopamine neurons suggest a possible link between mitochondrial calcium dysregulation, reduced mitochondrial function, and impaired neurophysiological activity which may ultimately contribute to the preferential vulnerability of dopamine neurons in PD.

## Discussion

It is essential to understand the order of appearance of pathophysiological phenotypes in neurodegeneration to develop disease modifying interventions effective in an early therapeutic window. Here we describe a pathophysiological timeline in which calcium release and store content deficits in mitochondria cause mitochondrial stress and a reduced ability to produce ATP. The resulting decrease in calcium and ATP leads to decreased spontaneous neuronal activity (measured by sEPSC) which ultimately compromise neuronal function and contributes to neuronal vulnerability.

In recent years, changes in electrophysiological activity as an early disease marker have been the focus of investigation within the PD field.^52^^,^^53^ Here we observed a decreased spontaneous electrophysiological activity after 70 days of neuronal maturation, a phenotype not previously described in a *GBA-*PD model. Electrophysiological dysfunction and aberrant activity have been described in both PD mouse models and human patients, occurring before the development of motor phenotypes, but coincident with cognitive perturbations.^54^^,^^55^^,^^56^ These data support the concept that neuronal *dysfunction* precedes *degeneration* in disease progression. Indeed, alterations to excitability before neuronal death is seen in other neurodegenerative disorders, for instance in Huntington’s disease and amyotrophic lateral sclerosis.^57^^,^^58^ The previously-undescribed synaptic phenotype we present here is consistent with the growing concept that perturbations in neuronal activity represent an early aspect of neurodegeneration.

The decreased activity we observe in *GBA-N370S* iPSC-dopamine neurons could represent a normal neuronal plastic adaptation in response to persistent decreased calcium levels. This would likely result in alterations in ion channel expression or synapse number, changing the probability of release (P_r_). The function of unitary sEPSC is still unclear but is distinct from action potential generated neurotransmitter release^59^ and has been linked with synaptic development.^60^ The reduced activity displayed in *GBA-N370S* iPSC-dopamine neurons may therefore cause an alteration in neuronal development which may play a role in early stages of genetic forms of PD.

PLD1 is involved in regulating calcium and has functional overlap with Phospholipase C. However, evidence suggests that PLD1 has a more prominent role in neurons as it is localized to synapses,^61^ involved in BDNF signaling (a key dopaminergic neurotrophic factor^62^) regulating neurotransmitter release,^61^ and is involved with neuronal development.^63^ PLD1 is, therefore, a central component linking neuronal physiological calcium signaling with synaptic activity. The PLD1 protein has previously been implicated in PD pathogenesis. In cell lines, PLD1 enzymatic activity is depressed by the hallmark PD associate protein, α-synuclein.^64^ However, α-synuclein clearance was positively regulated by PLD1 activity.^65^ Although seemingly contradictory, these data do support the premise for a role of PLD1 in -PD pathophysiological mechanism.

Our data demonstrate that *GBA-N370S* iPSC-dopamine neurons also have significantly reduced levels of iPLA2. iPLA2 is encoded by the *PARK14* gene and pathogenic mutations in the gene decrease calcium influx into intracellular stores, hindering calcium replenishment.^47^ Reduced iPLA2 expression in *GBA-N370S* iPSC-dopamine neurons suggests that calcium store replenishment is diminished, consistent with our findings that calcium signaling is strongly perturbed in *GBA-*PD. Our data show mitochondrial calcium signaling deficits revealed by either ionomycin or CCCP treatment. We propose that this deficit may be because of perturbed calcium release mechanisms, or a reduction in organelle content. The reduction in PLD1 and iPLA2 expression indicates that organelle calcium release and content are both impaired in *GBA-N370S* cellular phenotypes, although the exact mechanism by which the GCase mutation drives these changes in calcium biology is currently unclear.

Previous work by Schöndorf et al.^6^ in *GBA-L444P* iPSC-dopamine neurons showed an increased release of calcium from the ER.^6^ There are a number of methodological differences between their study and ours which mean the results cannot be directly compared. For example, we used treatment with CPA, a SERCA pump inhibitor, to measure ER calcium leak, whereas Schöndorf et al. used caffeine, a RyR2 agonist which induces acute organelle calcium-dependent release. No changes in ER stress proteins, nor of calreticulin levels, were reported by Schöndorf et al.^6^ Despite these methodological and phenotypic differences, as well as the different *GBA* mutations studied, the implication is clear that calcium dysregulation is a central pathophysiological aspect of *GBA-*PD.

Mitochondrial dysfunction is well established in genetic and sporadic PD^66^ and our work adds detailed new mechanistic information. In our *GBA*-PD model, mitochondrial function is impaired from an early stage of neuronal maturity and, as age is the major risk factor for PD, may contribute to the death of vulnerable dopaminergic neurons in the aging brain. Cell death in *GBA*-PD driven by dysfunctional mitochondria has been recently proposed to result from loss of GCase function. Both mitochondrial calcium release and mitochondrial membrane potential were decreased in a conditional knock-out *Gba* mouse model of Gaucher’s disease,^67^ a lysosomal storage disorder caused by homozygous GCase mutations, resulting in a reduced ATP:ADP ratio. Here, we confirm mitochondrial calcium dysregulation and then demonstrate a bioenergetic deficit in ATP production through reduced OCR in *GBA*-PD, likely driven by the decrease in Δψm. Furthermore, we also show the effect of energetic failure on the electrophysiological activity and synaptic function of the dopamine neurons vulnerable in PD.

The reduction in mitochondrial calcium which underlies these phenotypes may be caused by reduced calcium flux between the ER and the mitochondria. The ER is a major source supplying calcium to mitochondria after IP3R3 receptor stimulation and subsequent voltage dependent anion channel (VDAC) calcium uptake at ER-mitochondrial associated membranes (MAMs).^68^ We propose that reduced calcium flux between these two organelles is as a result of decreased MAM formation because of decreased proximity of ER and mitochondria in *GBA-N370S* iPSC-dopamine neurons. This is consistent with the disruption of ER-MAM function being a common pathological mechanism in PD.

Recent work has shown that synaptotagmin-1 is important for spontaneous release of neurotransmitter in general^51^ and specifically modulates spontaneous rather than activity-driven dopamine release from neurons.^69^ Given that we find changes specifically in spontaneous rather than evoked activity in *GBA-N370S* iPSC-dopamine neurons, we investigated synaptotagmin-1 expression in our model. We found a significant decrease in synaptotagmin-1 in *GBA-N370S* iPSC-dopamine neurons compared to controls in whole cell lysates, although we did not measure synaptotagmin 1 levels at the synapse or on synaptic vesicles. Synaptotagmin-1 drives neurotransmitter vesicle release as presynaptic calcium levels rise. Reduced levels of synaptotagmin-1 would result in less efficient neurotransmission which would be compounded by reduced calcium signaling. Synaptotagmin-1 therefore represents a possible mechanism by which pathologically depleted neuronal calcium signaling may manifest as aberrant synaptic activity, which might be tested by measuring synaptotagmin 1 levels at the synapse or on synaptic vesicles. Changes in synaptotagmin-1 do not rule out a possible role for other pre-synaptic proteins, nor does a lack of alteration to K_v_ or Na_v_ channel current rule out a role for other channels (BK, HCN etc) in the phenotypes observed.

The work we present utilizes the powerful platform of human iPSC-derived neuronal cultures, providing a human model system relevant to human neurodegeneration. However, the data presented here are from an *in vitro* monoculture cell model lacking the structural and multi-cellular complexity of a human brain. For this reason, any compensatory mechanisms likely induced by the aberrant phenotypes described may be missed. Further investigation in more complex co-cultures, organoids or animal models are vital in the future to fully understand the scope of the disease.

Overall, we report the first description of synaptic dysfunction in a *GBA*-PD model. Although other PD models exhibit altered dopamine content or release, these phenotypes need not reflect dysfunction at the synaptic level as alterations in neurotransmission may be because of changes in dopamine degradation or reuptake. Here, we report aberrant calcium homeostasis which may contribute to synaptic changes through a mechanism of depleted calcium and energy production because of disrupted mitochondrial function, and compromised bioenergetics, consistent with previous work. This occurs in conjunction with reduced levels of the synaptic vesicular protein, synaptotagmin-1, leading to a low efficiency of neurotransmitter release in response to rising levels of pre-synaptic calcium. Taken together, these data represent changes which may have a cumulative contributory effect to chronically reduce neuronal function, and lead to the late-stage neuronal susceptibility and subsequent degeneration of dopamine neurons in PD. The mechanisms described here represent, early pathological alterations which could potentially be targeted in *GBA-*PD patients to provide an early intervention before neuronal death.

## Limitations of the study

Although we found that critical players in calcium signaling were reduced in expression, we limited our work to looking at proteins either with a specific neuronal role (PLD1) or previously implicated in PD (iPLA2), and so our analysis is therefore by no means exhaustive. Other proteins may also contribute to the calcium dyshomeostasis and may represent viable targets. In addition, although the combination of approaches used focused on providing evidence for deficits in mitochondrial calcium stores, other cellular stores and organelles, such as lysosomes, may also be affected. It is expected that such changes would also impact disease relevant pathways.

## STAR★Methods

### Key resources table

**Table:** 

REAGENT or RESOURCE	SOURCE	IDENTIFIER
**Antibodies**		

Map2	Abcam	RRID:AB_2138147
Homer1	Synaptic Systems	RRID:AB_887730
Synapsin I/II,	Synaptic Systems	RRID:AB_1106784
PLD1	Cell Signaling	RRID:AB_2172256
iPLA2	Millipore	RRID:AB_310408
Synaptotagmin-1	Cell Signaling	RRID:AB_2798510
Tyrosine Hydroxylase	Millipore	RRID:AB_90755
IP3R3	Millipore	RRID:AB_571029
VDAC1	Abcam	RRID:AB_443084

**Biological samples**		

NHDF	EBISC	University of Oxford
SFC067-03	EBISC	STBCi105-A
SFC840-03	EBISC	STBCi026-D
SFC065-03	EBISC	STBCi057-A
SFC856-03	EBISC	STBCi298-A
JR053	EBISC	UOXFi005-A
SFC086-03	EBISC	STBCi052-B
SFC156-03	EBISC	STBCi101-A
OX1	EBISC	UOXFi004-B
SFC068-03	EBISC	STBCi106-A
MK082	EBISC	UOXFi002-A
SFC848-03	EBISC	STBCi042-A
MK088	EBISC	UOXFi003-A
SFC871-03	EBISC	STBCi084-B
MK071	EBISC	UOXFi001-B

**Chemicals, peptides, and recombinant proteins**		

FURA-2 QBT	Molecular Devices	R8198
CCCP	Sigma	C2759
FCCP	Sigma	C2920
CPA	Sigma	C1530
ROCK inhibitor (Y27632 dihydrochloride)	Bio-Techne	Cat#1254
GDNF	Peprotech	Cat#450-10
TGFb3	Peprotech	Cat#100-36E
DAPT	Abcam	Cat#ab120633
Ascorbic acid	Sigma	Cat#A4544
LDN-193189	Sigma	Cat#SML0559
SB-431542	Bio-Techne	Cat#1614
SHH C24II	Bio-Techne	Cat#1845-SH-500
Purmorphamine	Bio-Techne	Cat#4551/10
FGF8a	Stratech	Cat#16124-HNAE-SIB
CHIR-99021	Bio-Techne	Cat#4423
BDNF	Peprotech	Cat#450-02
(db)-cAMP	Sigma	Cat#D0627
hESC-qualified Matrigel	Corning	Cat#354277

**Oligonucleotides**		

Primers for PLD-1 KD	This paper	N/A
Primer: Scrambled control Forward: 5′-CACCGGCACTACCAGA GCTAACTCA-3′	This paper	N/A
Scrambled control Reverse: 5′AAACTGAGTTAGCTCTGGTAGTGCC-3′	This paper	N/A
PLD1 gRNA1 Forward: 5′-CACCGACAGCTATAGACATGCTCGG-3′	This paper	N/A
PLD1 gRNA1 Reverse: 5′-AAACCCGAGCATGTCTATAGCTGTC-3′	This paper	N/A
PLD1 gRNA2 Forward: 5′-CACCGGTGAGCCCACAAATAGACGG-3′	This paper	N/A
PLD1 gRNA2 Forward: 5′-CACCGGTGAGCCCACAAATAGACGG-3′	This paper	N/A
KO confirmation primer set:	This paper	N/A
5′-CCTGCTTTCTTGATGTTCTTTGC-3′	This paper	N/A
5′-GACTGCCTTGACAGGCTTAGA-3′	This paper	N/A

**Recombinant DNA**		

TLCV2	Addgene	RRID:Addgene_87360
psPAX2	Addgene	RRID:Addgene_12260
pMD2.G	Addgene	RRID:Addgene_12259

**Software and algorithms**		

Prism 8	Graphpad	https://www.graphpad.com/scientific-software/prism/
pclamp	Molecular Devices	https://www.moleculardevices.com/
Harmony	Perkin Elmer	n/a
Seahorse Wave	Agilent	https://www.agilent.com/en/product/cell-analysis/real-time-cell-metabolic-analysis/xf-software

### Resource availability

#### Lead contact

Further information and requests for resources and reagents should be directed to and will be fulfilled by the Lead Contact Richard Wade-Martins (richard.wade-martins@dpag.ox.ac.uk).

#### Materials availability

iPSC lines used as part of this study are available from the authors. Please contact the lead contact for access/more details. This study did not generate new unique reagents.

### Experimental model and subject details

#### iPSC stem cell lines

Participants were recruited to the Discovery clinical cohort through the Oxford Parkinson’s Disease Center and gave signed informed consent to mutation screening and derivation of iPSC lines from skin biopsies (Ethics committee: National Health Service, Health Research Authority, NRES Committee South Central, Berkshire, UK, REC 10/H0505/71). All the patients included in our study are European Caucasian and fulfilled UK Brain Bank diagnostic criteria for clinically probable PD at presentation.^70^

#### Generation of iPSC derived dopaminergic neurons

Neuronal differentiations^7^ were performed using induced pluripotent stem cells (iPSCs) derived from dermal fibroblasts obtained from donors of the Oxford Discovery Cohort (Table of iPSC lines used). Differentiation Basal media are as follows (Life Technologies unless stated otherwise): KO DMEM KSR = Knockout DMEM, Knockout serum replacement, 1X non-essential amino acids, 2 mM L-glutamine, 10 μM 2-mercaptoethanol (Sigma); NNB = Neurobasal medium, 0.5X N2 supplement, 0.5X B27 supplement, 2 mM L-glutamine; NB = Neurobasal medium, 1X B27 supplement, 2 mM L-glutamine. For differentiation factors added to basal media are as follows: 100 nM LDN-193189 (Sigma), 10 μM SB-431542 (Tocris Bioscience), 100 ng/mL recombinant sonic hedgehog C24II (R&D Systems), 2 μM purmorphamine (Calbiochem), 100 ng/mL fibroblast growth factor 8a (R&D Systems), 3 μM CHIR-99021 (Tocris Bioscience), 20 ng/mL brain-derived neurotrophic factor (Peprotech), 20 ng/mL glial cell line-derived neurotrophic factor (Peprotech), 1 ng/mL transforming growth factor β3 (Peprotech), 10 μM DAPT (abcam), 200 μM ascorbic acid (Sigma), 500 μM dibutyryl cAMP (Sigma). Medium containing differentiation and neurotrophic factors was fully changed every two days with half change every other day until day 20. Cells were then dissociated with StemPro Accutase (Life Technologies) and re-plated onto Geltrex at 600,000 cells/cm^2^ for coverslips and half area 96 well plates, and 300,000 cells/cm^2^ in 6 well plate. Cultures were treated at day 22 with 1 μg/mL mitomycin C in NB medium for 1 h to remove proliferating cells and washed with neurobasal medium before returning to fresh maturation medium. Media was subsequently changed every 3–4 days for the remaining period of maturation up to the respective timepoints of 35 and 70+ DIV. iPSC neuronal cultures were maintained at 37°C, 5% CO_2_. Cells were shown to be TH positive and differentiate similarly between control and GBA mutation lines (Figure S1). Electrophysiological characterisation illustrated large A-type K_v_ channel current, as well as a hyperpolarising sag in current clamp mode indicative of functional HCN channel expression (Figure S2). Both of these are indicative of nigral neurons. Finally, regular and spontaneous action potential persisted in the presence of network activity inhibition providing evidence of pacemaker activity, hallmark to SNpc neurons.

#### Table of iPSC lines used

Control and Patient iPSC lines used in the study from Oxford Parkinson’s Disease Center (OPDC) Discovery cohort.

**Table:** 

Cell line	Donor ID	PMID	iPSC clone	Genotype	Age & gender	Publication	hPSCreg ID for QC data
Ctrl 1	NHDF	24586273	1	wt	44 F	Hartfield et al. 2014^29^	
Ctrl 2	SFC067-03	30503143	1	wt	72 M	Lang et al. 2019^25^	STBCi105A
Ctrl 3	SFC840-03	26905200	1	wt	67 F	Fernandes et al. 2016^4^	STBCi026D
Ctrl 4	SFC065-03		3	wt	65 M	This manuscript	STBCi057-A
Ctrl 5	SFC856-03	28827786	4	wt	78 F	Hanseler et al. 2017^71^	STBCi298-A
Ctrl 6	JR053	30503143	1	wt	68 M	Lang et al. 2019^25^	UOXFi005-A
Ctrl 7	SFC086-03		2	wt	56 F	This manuscript	STBCi052-B
Ctrl 8	SFC156-03	30503143	1	wt	75 M	Lang et al. 2019^25^	STBCi101-A
Ctrl 9	OX1	23951090	18	wt	36 M	van Wilgenburg et al. 2013^72^	UOXFi004-B
Ctrl 10	SFC068-03		1	wt	67 M	This manuscript	STBCi106-A
GBA 1	MK082	36870058	31	N370S/wt	51 M	Bogetofte et al. 2023^73^	UOXFi002-A
GBA 2	SFC848-03	30503143	2	N370S/wt	68 M	Lang et al. 2019^25^	STBCi042-A
GBA 3	MK088	26905200	1	N370S/wt	46 M	Fernandes et al. 2016^4^	UOXFi003-A
GBA 4	SFC871-03		04	N370S/wt	70 F	This manuscript	STBCi084-B
GBA 5	MK071	26905200	3	N370S/wt	81 F	Fernandes et al. 2016^4^	UOXFi001-B

**Table of iPSC lines used.** All headings in the Table are denoted in bold. The following abbreviations are used in the Table:

Ctrl: Healthy Control donor.

GBA: Glucocerebrosidase mutation carrying donor.

F: Female.

M: Male.

wt: Wild Type.

N370S: Patient carrying *GBA N370S* mutation

PMID: Pubmed Identification.

### Method details

#### Electrophysiological recordings

Whole cell voltage-clamp recordings were performed on iPSC derived dopaminergic neurons at various stages of *in vitro* development, with the majority of recording being performed at 70+DIV which was defined as electrophysiologically mature. As described previously^74^ approximately 2 min after obtaining whole-cell configuration passive cellular properties were determined using the membrane test function. Recordings were conducted at in a heated (30°C) extracellular solution (ECS) containing (in mM unless stated): 167 NaCl, 2.4 KCl, 1 MgCl2, 10 glucose, 10 HEPES, 2 CaCl2, pH 7.4, ∼300 mOsm. Glass electrodes (resistance 4–7MΩ) for recordings were filled with intracellular potassium gluconate solution containing (in mM unless stated): 140 K-Gluconate, 6 NaCl, 1 EGTA, 10 HEPES, 4 MgATP, 0.4 Na_3_GTP, pH7.3, ∼290 mOsm.

Electrophysiological data acquisition, used a Multiclamp 700B amplifier and digidata 1550A and ClampEx 6 software. Data were filtered at 2 kHz, digitized at 10 kHz and tolerance for acceptable data was set prior to recordings as a series resistance (Rs) < 30 MΩ whist uncompensated. In addition, a tolerance cut-off of a ΔRs and/or ΔRm was set as <10%. Data was analyzed using Clampfit 10 (Molecular devices).

#### Immunocytochemistry

iPSC derived neurons were grown in a black 96 well half area plates with transparent bottom (Greiner) and fixed in the plate with 4% paraformaldehyde (PFA) for 5 min. Cells were permeabilised with 0.05% Saponin for 20 min and blocked for 30 min in PBS containing 10% goat serum at RT, before incubation with primary antibodies overnight at 4°C in PBS containing 0.1% Tween and 1% goat serum. Antibodies used as follows: Map2 (Abcam, #AB92424, 1:1000), Homer1 (Synaptic Systems, #160003, 1:500) and Synapsin I/II (Synaptic Systems, #106004, 1:500). AlexaFluor-conjugated secondary antibodies were incubated for 1 h at RT in PBS containing 0.1% Tween. Nuclear DNA was stained with DAPI (Thermo Fisher Scientific) for 5 min at RT. Images were captured by the Opera Phenix Plus High Content Screening System (Perkin-Elmer).

#### Ratiometric calcium flexstation recordings

Neurons on half area 96 well plates had 50% media removed and replaced with FURA-2 QBT made up in Hanks Balanced Salt Solution without calcium and without magnesium. Cells were left to incubate for 1 h at 37°C. Plates were loaded into the Flexstation 3 and the fluorescence calcium response to pharmacological stimulation (Ionomycin working concentration: 5 μM; CCCP working concentration: 20 μM; and CPA working concentration: 45 μM) was recorded after a 30-s baseline. Intracellular calcium was detected by excitation at 380 nm and 340 nm for bound and unbound FURA-2 QBT, respectively, with emission at 510 nm. Recordings were made every 4 s. All experiments ended with the application of ionomycin stimulation as a confirmatory positive control of possible calcium release. Ionomycin was applied 2 min post CCCP or CPA. All experiments were concluded 90 s post ionomycin application. Data was exported and analyzed on excel calculating the ratio of cytosolic calcium via 380/340. Response values to each compound were evaluated as the delta between the baseline and the response following injection. Baseline values were calculated as the average of all recordings before the first injection. Response values were evaluated by the average of 3–4 points around the peak. All results were then plotted on Graphpad PRISM and an independent-samples Student’s *t* test was conducted to compare compound responses between control and GBA cells.

#### Calcium measurements by genetically-encoded calcium indicator (GECI) imaging

The EF1a-GCEPIAer construct was obtained by subcloning GCEPIAer from the pCAG G-CEPIA1er plasmid (a gift from Franck Polleux (Addgene plasmid # 105012) downstream of an EF1a promoter in the Ef1a-mScarlet plasmid (a gift from Michael Ward) by EcoRI-HF and NheI-HF digestion and replacing mScarlet with GCEPIAer.

For ER calcium imaging, iPSC-dopamine neurons were transduced with GCEPIAer in a lentiviral construct at Day 20 and imaged at Day 35. The GECI was imaged using the FITC channel with a 20X water objective using an INCELL 6000 imager. The imaging was carried out in HBSS at 37°C for 64 s, with CPA injected at 16 s to a final concentration of 90 μM. The images were analyzed using the Columbus software.

#### Preparation of cell extracts

Cells were extracted with cold PBS and centrifuged at 500 × *g* for 5 min. Cell pellets were snap frozen and stored at −80°C until needed. Frozen cell pellets were lysed in 150 μL RIPA buffer (TRIS 1 M PH8, iGEPAL, Sodium Deoxy 10%, protease inhibitor cocktail (Sigma, 1 tablet/10 mL), phosSTOP (Sigma, 1 tablet/10 mL)) and briefly sonicated on ice (15 Amp, 10 s). The lysates were then centrifuged 20 min at 4 °C at full speed. Protein concentrations were estimated using the Pierce BCA protein assay kit (ThermoFisher) following the manufacturer’s instructions. The standard curve was built using a Bovine serum albumin standard. The absorbance was read on the Pherastar (BMG Labtech) at 562 nm.

#### SDS-PAGE and western blots

Extracted samples were diluted in homemade 6x Laemmli Buffer (for 10 mL: 20% SDS, 30% β-mercaptoethanol, 60% glycerol, 0.012% Bromophenol Blue, 375 mM Tris pH 6.8) and boiled at 90°C for 5 min (the exception were samples for PLD1 assessment which were left unboiled). Samples were loaded on a 4–15% gradient gel (Bio-Rad) and separated via SDS-PAGE (1 h, 120 mV) using a homemade running buffer (144 g glycine, 30.3 g TRIS Base, 10 g SDS for 1 L of 10X buffer). Proteins were then transferred onto a midi PVDF membrane using the High Molecular Weight protocol on the Trans-Blot Turbo Transfer System (Bio-Rad).

The membrane was blocked in TBS-Tween0.1%-Milk 5% for 1 h at room temperature and then incubated overnight at 4°C with the primary antibody in TBS-Tween0.1%-Milk 1%. For the scope of this paper, membranes were separately probed with the following antibodies: PLD1 (Cell Signaling, 3832S), iPLA2 (Merck Millipore, 07-169-I), Synaptotagmin-1 (Cell Signaling, 3347S). Each membrane was then washed 3∗10 min in TBS-Tween 0.1% and incubated for 1 h at room temperature with an HRP-conjugated secondary antibody (Bio-Rad) in TBS-Tween0.1%-Milk 1%. Finally, the blot was washed and imaged on the ChemiDoc-Touch (Bio-Rad) with ECL substrate (GE Healthcare). After a rapid wash in TBS-Tween 0.1%, the membrane was stained with an HRP-conjugated antibody directed toward β-actin (Bio-Rad) and imaged.

Densiometric analysis of the protein bands was performed using the volume analysis tool on ImageJ. Each measurement was normalised to actin prior to the comparison of protein abundance between control and *GBA-N370S* iPSC-dopamine neurons. Due to differentiation variability, controls were included in every “batch differentiation” and GBA ratio was normalised to this where control was equal to 1.

#### Lentiviral vectors and plasmid construction

TLCV2 (Addgene #87360) was digested using the restriction enzyme BsmBI, dephosporylated and gel-purified. Protospacer sequences were appended in the form of double stranded oligonucleotides with complementary sticky ends to the vector backbone (Scrambled control Forward Oligo 5′-CACCGGCACTACCAGAGCTAACTCA-3′, Scrambled control Reverse Oligo 5′AAACTGAGTTAGCTCTGGTAGTGCC-3′, PLD1 gRNA1 Forward Oligo 5′-CACCGACAGCTATAGACATGCTCGG-3′, PLD1 gRNA1 Reverse Oligo 5′-AAACCCGAGCATGTCTATAGCTGTC-3′, PLD1 gRNA2 Forward oligo 5′-CACCGGTGAGCCCACAAATAGACGG-3′, PLD1 gRNA2 Reverse Oligo 5′-AAACCCGTCTATTTGTGGGCTCACC-3′).

Lentiviral particles were produced by transfecting 5 μg of TLCV2 constructs together with packaging components (3.75 μg psPAX2 Addgene #12260 and 1.5 μg pMD2.G Addgene #12259) into human embryonic kidney cells (American Type Culture Collection) that were plated 24 h before transfection at the seeding density of 4 x 106 cells/T75 flask. Transfection in each flask was performed in the presence of 30 μL of Lipofectamine 2000 reagent (Invitrogen), according to the manufacturer’s instructions. Viral supernatants were harvested 48 h post transfection, passed through a 0.45 μm filter (Millipore) and frozen at −80°C until further use.

#### Lentiviral transduction for PLD1 knockdown

24 h before transduction, 2.5 x 10^5^ SH-SY5Y cells were seeded in 2 mL of complete medium into 6-well plates. Virus was titrated and the volume of virus used was calculated using an MOI of 0.7. Viral vectors were used to transduce cells in the presence of polybrene (8 μg/mL, Millipore). A full media change was performed 24 h post-transduction. Puromycin selection (1 μg/mL, Millipore) was started 24 h post viral transduction, and then gradually increased to 2 μg/mL for the following cell passages. Cas9-GFP expression was induced with Doxycycline (1 μg/mL) 48 h post transduction and was confirmed with fluorescence microscopy (EVOS). Knock out of PLD1 was confirmed using qPCR with the following primer set (5′-CCTGCTTTCTTGATGTTCTTTGC-3′, 5′-GACTGCCTTGACAGGCTTAGA-3′).

#### Cell culture

HEK293T cells were maintained in DMEM high glucose (Sigma) supplemented with 10% FBS (Life Technologies), 1% L-glutamine (2 mM, Life Technologies), 1% pen-strep (Life Technologies), Non-essential amino acids (10 mM; Gibco). SH-SY5Y cells were cultured in DMEM/F12 (1:1 1X, Life Technologies) supplemented with 10% FBS (Life Technologies), 1% L-glutamine (2 mM, Life Technologies) and 1% pen-strep (Life Technologies). All cells were incubated at 37°C with 95% air and 5% CO_2_.

##### Measurement of mitochondrial respiration

Oxygen consumption rate (OCR) was measured using an XF96e Extracellular Flux Analyzer (Seahorse Bioscience). Cells were seeded on XF96-well cell plates on day 20 and further matured until day 35 or 70 before analysis. On the day of the assay, medium was replaced with XF Base Medium (Seahorse Bioscience) supplemented with 10 mM Glucose (Sigma-Aldrich-Aldrich), 1 mM Sodium Pyruvate (Sigma-Aldrich-Aldrich) and 2 mM L-Glutamine (Thermo Fisher Scientific). Cells were then incubated at 37°C in a non-CO_2_ incubator for 1 h. Changes in oxygen consumption were measured following sequential injection of the ATP synthase inhibitor oligomycin (1 μM; Sigma-Aldrich), the mitochondrial uncoupler *p*-triflouromethoxyphenylhydrazone (FCCP 1 μM; Sigma-Aldrich), and the Complex I and III inhibitors Rotenone and Antimycin A (0.5 μM; Sigma-Aldrich). Values were normalized to total protein content of each well and analyzed according to the manufacturer’s guidelines.

##### Determination of mitochondrial membrane potential

To measure mitochondrial membrane potential (ΔΨm), cells were washed with Krebs buffer (145 mM NaCl, 5 mM KCl, 10 mM HEPES, 1 mM MgCl2, 1 mM CaCl2, 5.6 mM glucose and pH 7.4/NaOH) and loaded with JC-10 (5 μM; AAT Bioquest, Inc.) at 37°C for 30 min. Green fluorescence (depolarization) was monitored at 529 nm and red fluorescence (polarized) at 590 nm on a multi-mode plate reader PHERAstar FSX (BMG Labtech). To establish that the JC-10 signal was indicative of ΔΨm, experiments were terminated inducing a maximal mitochondrial depolarization by addition of carbonyl cyanide 3-chlorophenylhydrazone (CCCP 10 μM; Sigma-Aldrich).

##### Proximity ligation assay

To measure ER-mitochondrial proximity,^75^ cells were fixed with 4% PFA and stained for TH (1:500 Millipore). Cells were then blocked for PLA and incubated with primary antibodies against IP3R3 (1:500 Merck) and VDAC1 (1:500 Abcam) ON. After washing PLA probes anti-mouse and anti-rabbit were used to complete the PLA reaction as per manufacturer’s instructions (Merck Sigma). DAPI was used as a nuclear stain and the puncta from 25 neurons per line and differentiation were counted blindly to determine average puncta per TH neuron.

### Quantification and statistical analysis

#### Statistical analysis

For the analysis of Seahorse assay and JC 10 assays we identified the difference between Conditions A and B, using linear mixed modeling. Because the data of both conditions (A and B) were drawn from different differentiations, we assumed ‘differentiation’ as the random effect in our model, whereas the ‘condition’ was represented as the fixed effect. Assuming the effect of differentiation on the response variable (Data) same in every group (i.e. random intercepts model), the model was formulated as M0: Data ∼ Condition + (1 | Differentiation). We further assumed the differentiation to have a different effect for each group and formulated our random slopes model as M1: Data ∼ Condition + (1 + Condition | Differentiation). We compared both models using the likelihood ratio test (LRT), the Akaike information criterion (AIC), and the Bayesian information criterion (BIC). Since the random slopes model was not providing significant improvement over random intercepts model in terms of LRT, AIC, and BIC, we preferred to continue our analysis with the random intercepts model (M0). Data were analyzed with the use of R statistical software version 4.0.0. Single cell electrophysiological analysis was performed using Prism6 software (Graphpad, Inc.). Direct comparisons were made by Student’s *t*-test (two-tailed, herein, *t*-test). Western blot analysis was performed using Mann-Whitney U Test. All significances are indicated by asterisks: ∗p < 0.05, ∗∗p < 0.01, ∗∗∗p < 0.001, ∗∗∗∗p < 0.0001. All of the statistical details of experiments can be found in the Figure legends.

## Data Availability

Data• All data reported in this paper will be shared by the lead contact upon request. Code• This paper does not report original code. • Any additional information required to reanalyze the data reported in this paper is available from the lead contact upon request.
