# Supplementary material for: Calcium dysregulation combined with mitochondrial failure and electrophysiological maturity converge in Parkinson’s iPSC-dopamine neurons
Source: iScience. 2023 Jun 7;26(7):107044. doi: 10.1016/j.isci.2023.107044 (PMC10329047; doi:10.1016/j.isci.2023.107044)
Supplement: Document S1. Figures S1–S4 [file mmc1.pdf]

iScience, Volume *26*

Supplemental information

Calcium dysregulation combined with mitochondrial failure and electrophysiological maturity converge in Parkinson's iPSC-dopamine neurons

Dayne A. Beccano-Kelly, Marta Cherubini, Yassine Mousba, Kaitlyn M.L. Cramb, Stefania Giussani, Maria Claudia Caiazza, Pavandeep Rai, Siv Vingill, Nora Bengoa-Vergniory, Bryan Ng, Gabriele Corda, Abhirup Banerjee, Jane Vowles, Sally Cowley, and Richard Wade-Martins

SUPPLEMENTARY INFORMATION

Beccano-Kelly et al. Supplementary Figure 1

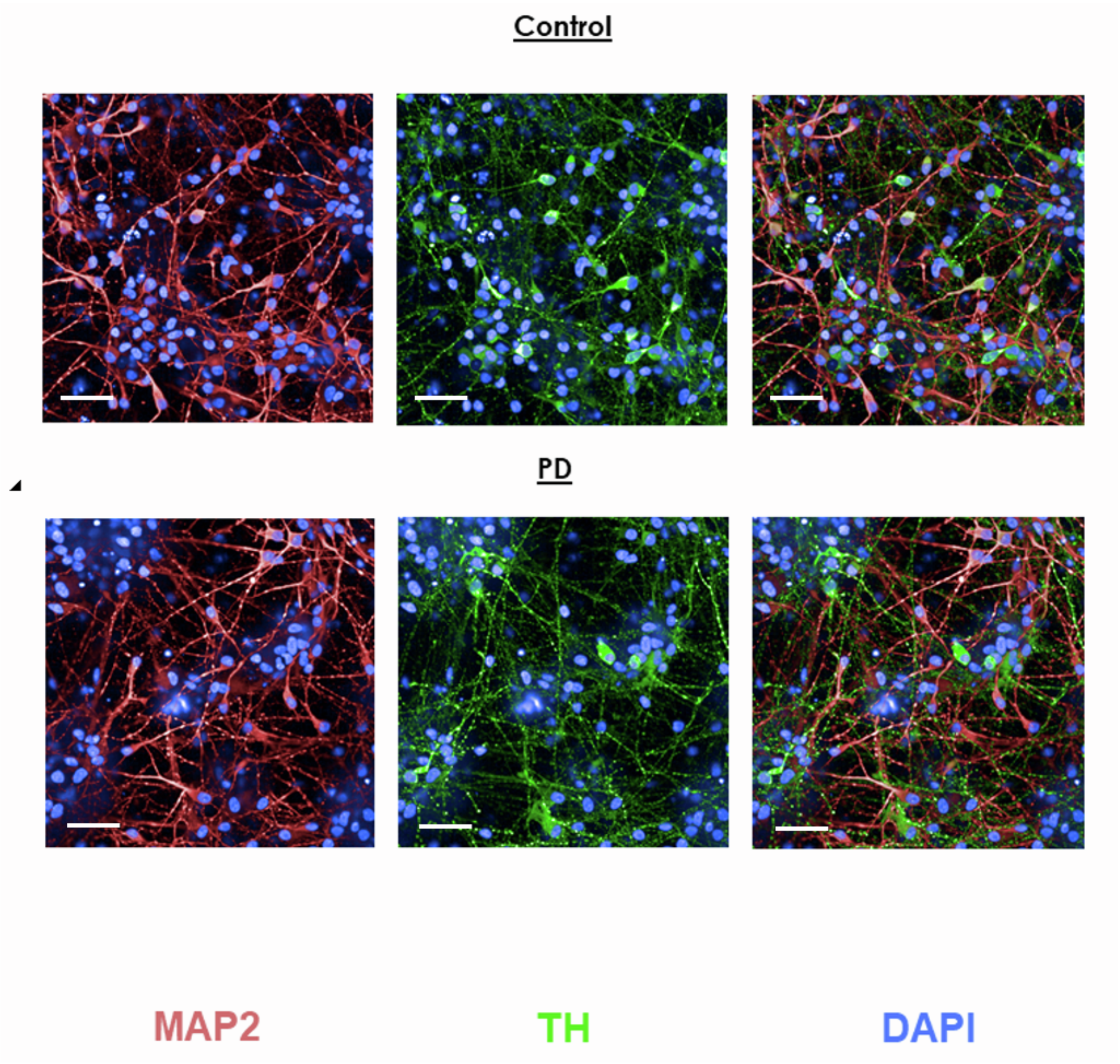

Fig. S1.
Equivalent differentiation of control and *GBA-N370S* iPSC-derived dopamine neurons.

Related to Figure 1 and STAR Methods

Both control (**Upper panels**; Ctrl 2) and *GBA-N370S* (**Bottom panels**; GBA 4) iPSC-dopamine neurons stained positively for TH, MAP2 and DAPI at 35 DIV with no morphological or numerical difference between the lines. Scale Bar = 50 µm.

Beccano-Kelly et al Supplementary Figure 2

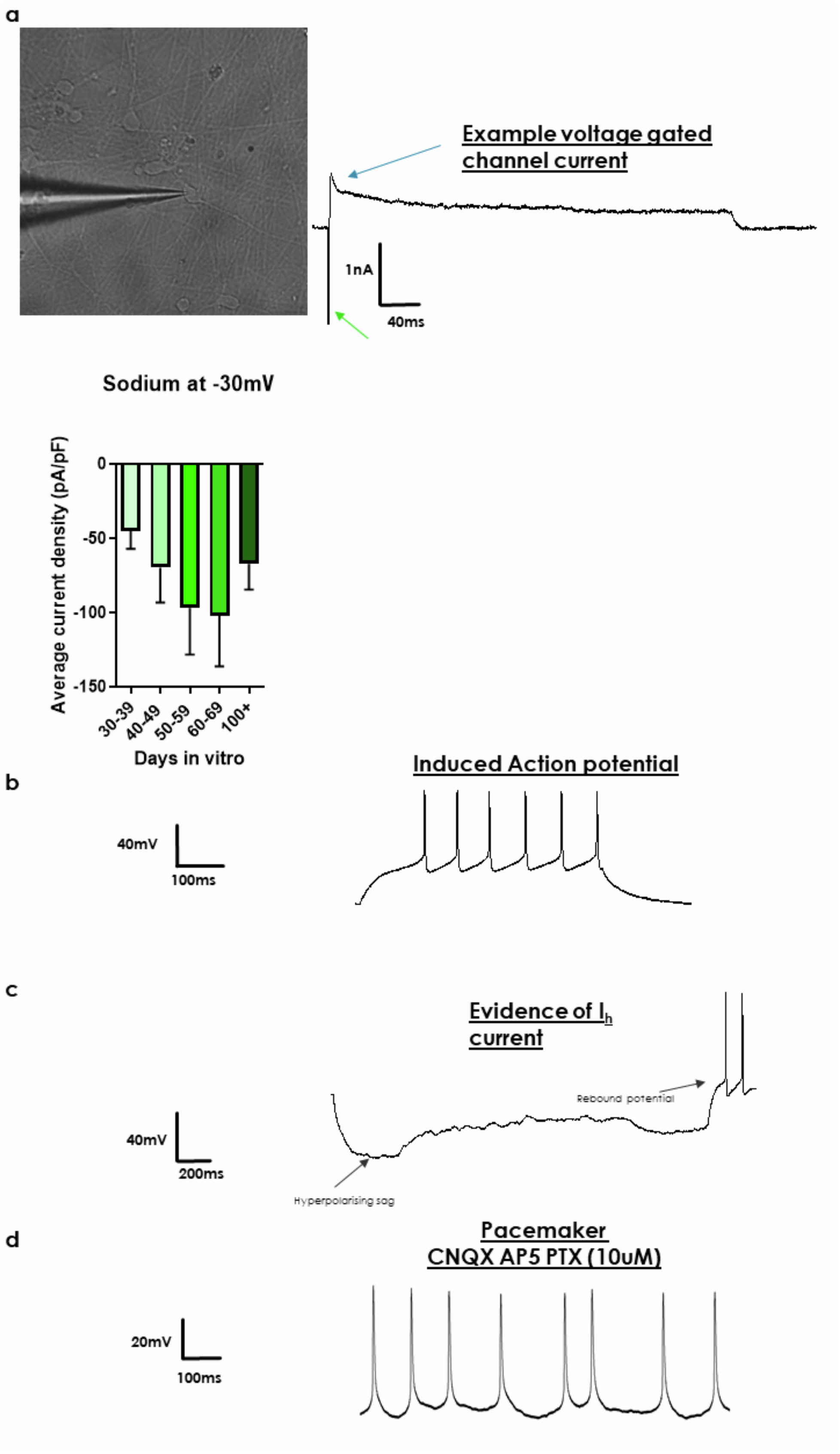

Fig. S2.

Electrophysiological measurements of differentiated iPSC-dopamine neurons showed substantia nigra-like properties. Related to Figure 1 and STAR Methods.

a. ***Upper Left:*** Brightfield Image of iPSC dopaminergic neuronal culture undergoing electrophysiological recording. Scale Bar = 50µm. ***Right:*** Example voltage gated current trace displaying large A-type potassium channel current. ***Lower left:*** Current density of voltage gated sodium channels over time shows increasing amplitude over time peaking at approximately 50-59 DIV. All data represented as mean +/- SEM**.** **b.** Example trace of induction of regular mature action potential train. **C.** Example trace showing hyperpolarizing sag caused by I_h current and subsequent "rebound" potentials. **D.** Example trace of pacemaker activity in the presence of a network activity inhibitor cocktail (cyanquixaline, (2R)-amino-5-phosphonovaleric acid and Picrotoxin, all at 10 µM working concentration) proving intrinsic spontaneous activity.

Beccano-Kelly et al Supplementary Figure 3

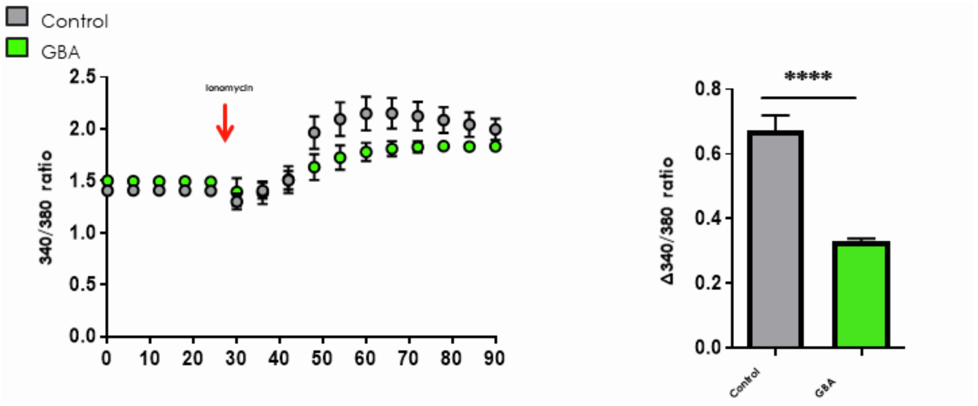

Fig. S3.

A deficit in ionomycin-induced calcium store release is evident at early stages of differentiation. Related to Figure 2.

Left: Example calcium release from Ctrl line 1 and *GBA-N370S* line 3 iPSC-dopamine neurons from 1 differentiation at 35 DIV, evoked by ionomycin (red arrow) as measured by ratiometric fluorescent dye Fura2 on Flexstation 3. ***Right:*** Delta between baseline and average response representing magnitude of change in somatic calcium in response to ionomycin addition ($p<0.0001$ unpaired *t*-test with Welch's correction). All data represented as mean +/- SEM **** $p < 0.0001$.

Beccano-Kelly et al Supplementary Figure 4

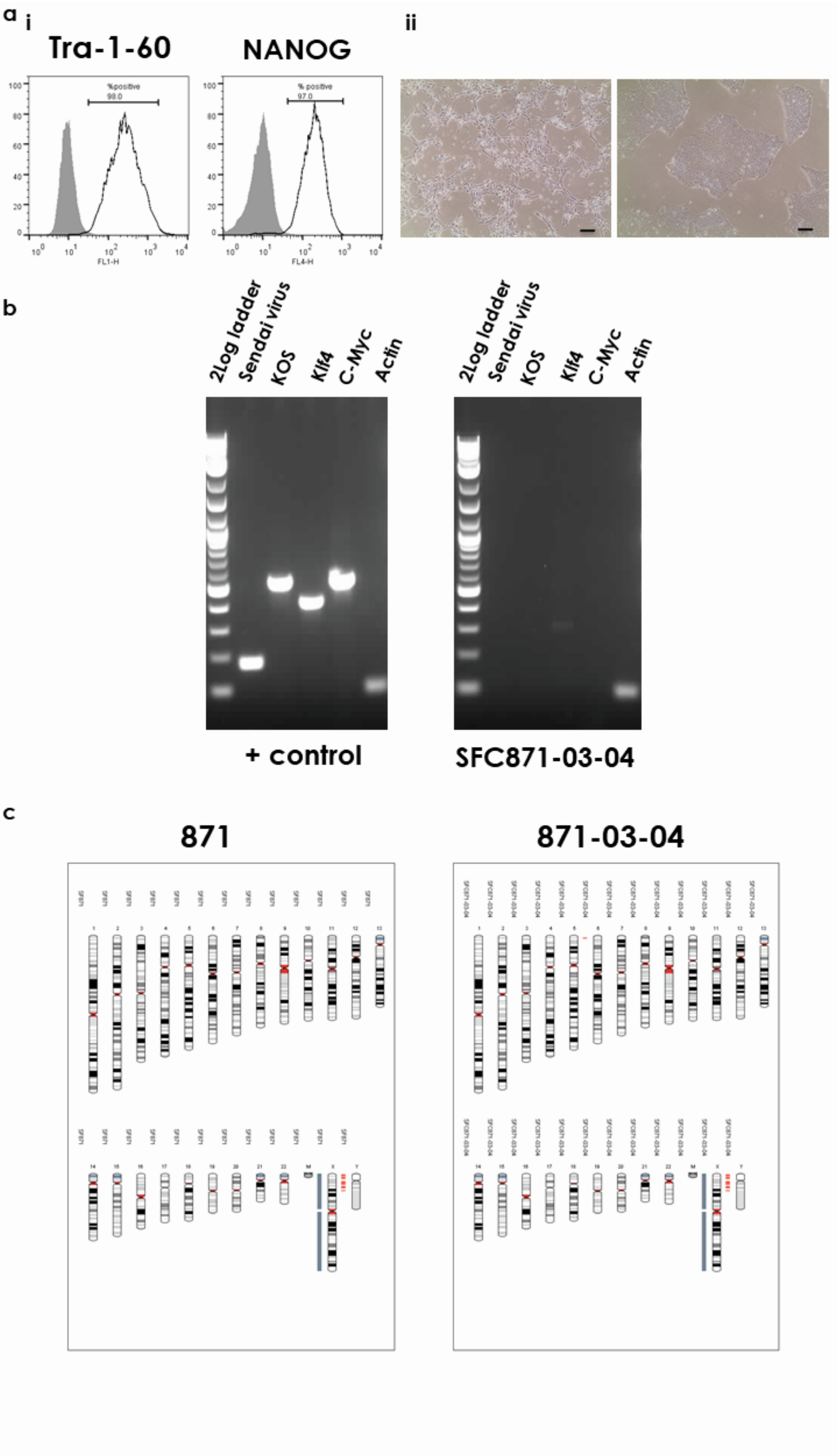

Fig. S4.
Generation of GBA(N370S) PD iPSC line from patient fibroblast 871-03-04. Related to Figures 2, 3 and 4 and Table 1.

a i: PD iPSC lines express expected pluripotency proteins, as shown by FACs for Tra-1-60 and Nanog (grown feeder-free); open black plot represents antibody, filled grey plot represents isotype control; **ii:** Panel shows the expected iPSC colony morphology both 24 hours post thaw (left) and 96 hours post thaw (right); scale bar = 100 µm. Images show the expected clustering of cells over time and a high nucleus to cytoplasm ratio by phase-contrast microscopy. **b.** Cytotune Sendai virus clearance in iPSC lines by RT-PCR. Blots show that iPSC lines show the correct size band for β-actin 92 bp for both the control (+ control, fibroblasts infected with Cytotune 5 days previously) and *GBA-N370S* line 871-03-04 reprogrammed line. No bands corresponding to the reprogramming virus PCR product sizes, specifically Sendai backbone 181 bp; S, Sox2 451 bp; K, Klf4 410 bp; M, c-myc 532 bp; O, Oct-4 483 bp were shown in the 871-03-04 line. **c.** Genome integrity was assessed by Illumina Human CytoSNP-12v2.1 or OmniExpress24 SNP array and karyograms produced using KaryoStudio software (Illumina). Amplifications (green), deletions (orange) and LOH regions (grey) are shown alongside the relevant chromosome (except that in females the X chromosomes are annotated with grey, and single-copy sex chromosomes are annotated red).
